# Building the Future of Aging: Investigating Strategies and Innovations in Master Plans on Aging

**DOI:** 10.1093/geroni/igaf122.140

**Published:** 2025-12-31

**Authors:** Lydia Manning, Carey Peerman

**Affiliations:** Scripps Gerontology Center, Oxford, Ohio, United States; Radford University, Radford, Virginia, United States

## Abstract

Master Plans on Aging (MPAs) are essential frameworks designed to address the challenges that older adults face and ensure that states have a comprehensive strategy for aging-related policies, services, and programs. This presentation explores the development and implementation of MPAs across five states: Colorado, California, Texas, Massachusetts, and Minnesota, and highlights the frameworks used, the roles of political leadership, and the multi-sector collaboration required to create such plans. This qualitative, exploratory study, with 25 stakeholder interviews, utilizes a grounded theory approach to investigate the strategies and innovations that have shaped the development and implementation of Master Plans on Aging (MPAs) in the five U.S. states with plans. The analysis reveals several commonalities and differences across states, as well as critical insights into the effectiveness of these plans. Furthermore, findings provide insight into the planning and stakeholder engagement processes, integration of these plans into state-level policies, and funding mechanisms that support their implementation. The presentation will discuss key innovations in aging policy, including creating age-friendly communities, affordable housing, and economic security for older adults, as well as successful models for service delivery and evaluation metrics. Participants will gain insights into the collaborative processes that have shaped these plans and learn best practices for overcoming aging, equity, and inclusion barriers. This session will provide a comprehensive overview of how MPAs can effectively improve the quality of life for older adults and offer a roadmap for developing similar initiatives across the U.S. and beyond.

